# Multi-Target Photoprotection by Taxifolin Against UVB-Induced Keratinocyte Injury Through UVB Filtration, ROS Scavenging and Transcriptomic-Proteomic Reprogramming

**DOI:** 10.3390/biom16030387

**Published:** 2026-03-04

**Authors:** Fangfang Chen, Yihan Cai, Jinxiong Wu, Nengzhen Fang, Fei Li, Hongtan Wu, Yu-Pei Chen

**Affiliations:** 1 The School of Public Health and Medical Technology, Xiamen Medical College, Xiamen 361023, China; 200700010183@xmmc.edu.cn (F.C.); caiyihan1@xmmc.edu.cn (Y.C.); wujinxiong@xmmc.edu.cn (J.W.); fangnengzhen@xmmc.edu.cn (N.F.); lifei1@xmmc.edu.cn (F.L.); wht@xmmc.edu.cn (H.W.); 2 Engineering Research Center of Natural Cosmeceuticals College of Fujian Province, Xiamen Medical College, Xiamen 361023, China

**Keywords:** taxifolin, UVB, ROS, JNK/p38 MAPK, multi-omics

## Abstract

Taxifolin, a natural flavonoid, consistently exerts cytoprotective effects against various oxidative stresses. In this study, we systematically evaluated its photoprotective efficacy and underlying mechanisms against ultraviolet B (UVB)-induced injury in human immortalized keratinocytes (HaCaT). Cell viability and apoptosis were assessed by MTT, fluorescence staining, and flow cytometry, while integrative transcriptomic and proteomic analyses were employed to identify core pathways and key mediators. Taxifolin exhibited antioxidant capacity comparable to that of ascorbic acid under identical in vitro radical-scavenging assays. Moreover, it displayed a strong absorption peak at 289 nm that overlaps the UVB spectrum (280–320 nm), enabling it to act as a chemical sunscreen. In UVB-challenged HaCaT cells, taxifolin markedly reduced intracellular reactive oxygen species (ROS) and attenuated JNK/p38 MAPK activation, as evidenced by Western blot, thereby breaking the ROS-MAPK vicious cycle. Multi-omics revealed that taxifolin was associated with attenuation of UVB-imposed G1/S arrest concomitant with restored Cyclin expression, while up-regulating MYC, FOXQ1, HMOX1 and AP-1 components c-Jun/c-Fos and thereby switching on a pro-survival transcriptional program. Consequently, apoptosis was suppressed and survival was significantly improved. Collectively, taxifolin integrated chemical filtration, ROS scavenging and signaling modulation to support a multi-target photoprotective network, which provides mechanistic insight into taxifolin-mediated cytoprotection and identifies candidate molecular nodes for further validation.

## 1. Introduction

Prolonged UVB exposure induces DNA damage and activates the DNA damage response, leading to cell cycle arrest and apoptosis. This arrest provides a crucial time for DNA repair; if the damage remains unresolved, cells may initiate programmed death to prevent mutation accumulation [1]. UVB triggers these apoptotic pathways through intrinsic and extrinsic mechanisms. The intrinsic, mitochondria-mediated pathway involves UVB-induced up-regulation of pro-apoptotic Bcl-2-associated X protein (Bax) and suppression of anti-apoptotic B-cell lymphoma 2 (Bcl-2) [2]. This disrupts mitochondrial membrane potential, leading to cytochrome c release and subsequent activation of the cysteine-aspartic protease (Caspase)-9/-3 cascade [3]. The extrinsic pathway is initiated throughup-regulationn of death receptors (cluster of differentiation 95 (CD95), tumor necrosis factor receptor (TNFR), TNF-related apoptosis-inducing ligand receptor (TRAIL-R)). Their cytoplasmic tails recruit Fas-associated death domain protein (FADD), rapidly cleaving pro-caspase-8 to active caspase-8, which in turn activates caspase-3 and executes the extrinsic apoptotic program [2]. Additionally, UVB-generated ROS creates oxidative stress, exacerbating cellular damage and mitochondrial dysfunction, thereby further promoting apoptotic signaling and cell death [4].

Taxifolin (dihydroquercetin, 2,3-dihydroquercetin) is widely distributed across plant species but varies greatly in concentration, with the *Larix* genus (Pinaceae) being the richest source. It also occurs naturally in fruits like apples (peel > flesh). Given its limited water solubility, extraction is commonly performed using 75–80% aqueous ethanol [5,6]. Taxifolin is a dihydroflavonol compound (C_15_H_12_O_7_, MW: 304.25 g/mol, CAS: 480-18-2) characterized by a saturated C2-C3 bond in the C-ring, which distinguishes it from the structurally related flavonol quercetin. The structure-activity relationship of taxifolin is defined by its pentahydroxyflavanone backbone. Specifically, the catechol moiety (3′,4′-dihydroxyl) on the B-ring and the 3-hydroxyl group on the C-ring are responsible for its potent radical-scavenging capacity [7]. Furthermore, the saturated C2-C3 bond distinguishes it from quercetin and confers enhanced chemical stability, although this structural modification results in a modest reduction in chemical antioxidant potency compared to its flavonol counterpart. Thus, taxifolin effectively scavenges free radicals (DPPH, ABTS, ·OH), chelates Fe^2+^ to inhibit the Fenton reaction, and suppresses ROS generation [7,8]. Research highlights its role in modulating oxidative stress, mitochondrial function, and key signaling pathways (mitogen-activated protein kinase (MAPK) and phosphoinositide 3-kinase (PI3K)/protein kinase B (Akt)) across disease contexts. In melanoma, it reduces ROS, inhibits proliferation/migration/invasion, and promotes cell apoptosis [9,10]. In liver ischemia–reperfusion injury, it enhances PINK1/Parkin-mediated mitophagy to alleviate damage [11]. In pancreatic cancer, it downregulates the HIF-1 pathway to achieve an anti-tumor effect [12]. Taxifolin also activates nuclear factor erythroid 2-related factor 2 (Nrf2) to protect against glucocorticoid-induced bone necrosis [13] and 5-fluorouracil-induced cardiotoxicity [14], while blocking MAPK/PI3K-Akt to exert antithrombotic effects [15]. Furthermore, it mitigates diabetic neuropathy by reducing ROS and inflammatory markers [16] and protects retinal pigment epithelial cells via the Nrf2 pathway, suggesting potential in age-related macular degeneration (AMD) treatment [17]. Overall, taxifolin functions as a multi-target redox regulator, influencing microenvironment and cell fate, and demonstrates context-dependent cytoprotective potential in oxidative stress models.

In addition, taxifolin protects primary human keratinocytes (NHEKs) and dermal fibroblasts (NHDFs) from UVA-induced oxidative damage by activating the Nrf2-antioxidant defense pathway [18]. This action upregulates enzymes like heme oxygenase-1 (HO-1), NAD(P)H quinone oxidoreductase 1 (NQO1), and catalase (CAT), counteracting glutathione depletion and DNA damage. Unlike UVA, UVB’s carcinogenicity stems largely from direct DNA damage and signaling dysregulation, particularly through the epidermal growth factor receptor (EGFR)-PI3K/Akt axis, which promotes cell survival and proliferation. Persistent activation of this pathway increases cancer risk. Recent studies demonstrate that taxifolin directly inhibits EGFR and PI3K kinase activity in JB6P^+^ mouse epidermal cells, significantly delaying UVB-induced skin carcinogenesis [19].

Despite the aforementioned findings highlighting the potential of taxifolin to mitigate UVB-induced skin damage, several critical dimensions remain unexplored. First, while taxifolin’s UVB absorption properties have been characterized spectroscopically, its chemical photofiltration efficacy has not been evaluated in human keratinocyte models. Second, although MAPK signaling is known to mediate UVB-induced damage, the integration of ROS-MAPK-cell cycle axis in taxifolin-mediated photoprotection remains undefined. Third, a comprehensive literature search revealed no prior study integrating RNA-seq and proteomic analysis in this context. Thus, we integrate transcriptomic and proteomic approaches to comprehensively characterize the signaling networks modulated by taxifolin. This study aims to identify the key molecular pathways through which taxifolin confers protection against UVB-induced injury in human skin cells, thereby providing a mechanistic rationale and potential therapeutic targets for the development of taxifolin-based photoprotective strategies.

## 2. Materials and Methods

### 2.1. Antioxidant Assays

#### 2.1.1. 2,2-Diphenyl-1-Picrylhydrazyl (DPPH) Radical-Scavenging Activity

DPPH (2.5 mg) was dissolved in ethanol and made up to 10 mL to obtain a 0.25 mg mL^−1^ stock solution. The stock was diluted with ethanol until the absorbance at 517 nm reached 0.8–1.0 to prepare the working solution. Taxifolin (purity ≥ 98%, Shanghai Aladdin Bio-Chem Technology Co., Ltd., Shanghai, China) and ascorbic acid (positive control) were dissolved in ethanol at graded concentrations. In a 96-well plate, 180 μL of DPPH working solution was mixed with 20 μL of sample solution. After 10 min incubation at room temperature in the dark, the absorbance at 517 nm was recorded in triplicate and the scavenging activity was calculated.

#### 2.1.2. 2,2′-Azinobis-(3-Ethylbenzothiazoline-6-Sulfonic Acid) (ABTS) Radical- Scavenging Activity

The ABTS working solution was prepared according to the manufacturer’s instructions (Beyotime Biotechnology Co., Ltd., Shanghai, China). Briefly, equal volumes of ABTS solution and oxidant reagent were combined and kept in the dark at room temperature for 12–16 h to generate the ABTS·+ stock. The stock was diluted with ethanol to an absorbance of 0.7 at 734 nm. Sample solutions (taxifolin or ascorbic acid) at graded concentrations were prepared. Each well received 180 μL of ABTS working solution and 20 μL of sample. After 5 min incubation in the dark, absorbance was read at 734 nm.

#### 2.1.3. Ferric Reducing Antioxidant Power (FRAP) Antioxidant Capacity

The FRAP working solution was freshly prepared by mixing 2,4,6-tripyridyl-s-triazine (TPTZ) diluent, TPTZ solution and assay buffer at 10:1:1 (*v*/*v*/*v*) as specified in the T-AOC Assay Kit (FRAP) (Beyotime Biotechnology Co., Ltd., Shanghai, China). A volume of 180 μL of working solution was added to each well, followed by 20 μL of taxifolin solution. After 20 min incubation in the dark, the absorbance at 593 nm was recorded. A standard curve was constructed with FeSO_4_·7H_2_O, and results are expressed as Fe^2+^ equivalents (mM).

### 2.2. 3-(4,5-Dimethylthiazol-2-yl)-2,5-Diphenyltetrazolium Bromide (MTT) Cell Viability Assay

HaCaT cells were purchased from the commercial source BeNa Culture Collection (BNCC, Xinyang City, China). HaCaT cells were seeded in 96-well plates and allowed to attach overnight at 37 °C in 5% CO_2_. Before treatment, the medium was replaced with serum-free Dulbecco’s modified Eagle medium (DMEM); cells were then exposed to serial concentrations of taxifolin or an equal volume of vehicle (ethanol) for 1 h or 12 h. After removing the supernatant, 90 μL of serum-free DMEM plus 10 μL MTT solution (5 mg mL^−1^) was added to each well and incubated at 37 °C for 4 h in the dark. The medium was aspirated, 150 μL dimethyl sulfoxide (DMSO) was added, and the plates were shaken for 10 min to fully dissolve the purple formazan crystals. Absorbance was read at 490 nm, and cell viability was calculated as: (A_sample/A_control) × 100.

### 2.3. Intracellular ROS Measurement

HaCaT cells were seeded in black 96-well plates and allowed to attach overnight at 37 °C under 5% CO_2_. The medium was then replaced with serum-free DMEM, and cells were treated with graded concentrations of taxifolin or an equal volume of ethanol vehicle for 1 h. Subsequently, the plates were exposed to UVB irradiation (312 nm, 225 mJ cm^−2^) using the Bio-Sun system (Vilber Bio Imaging, Collégien, France); a non-irradiated group was included in parallel. After irradiation, cells were immediately returned to the incubator for 1 h. For ROS detection, 2′,7′-dichlorodihydrofluorescein diacetate (DCFH-DA) was diluted to 10 µM in serum-free DMEM according to the manufacturer’s instructions (Beyotime Biotechnology Co., Ltd., Shanghai, China). Culture medium was removed, and 100 µL of the DCFH-DA working solution was added to each well. After 30 min incubation at 37 °C in the dark, cells were gently washed twice with serum-free DMEM to remove unbound probe. Fluorescence was measured with a microplate reader at 488 nm excitation/525 nm emission (Infinite 200Pro, Tecan, Männedorf, Switzerland) and is expressed as relative fluorescence units.

### 2.4. Western Blot Analysis

HaCaT cells were seeded in Petri dishes and allowed to attach overnight at 37 °C under 5% CO_2_. After replacement with fresh medium, cells were treated with graded concentrations of taxifolin or an equal volume of ethanol vehicle for 1 h, exposed to UVB irradiation (312 nm, 225 mJ cm^−2^) in a Bio-Sun system (Vilber Bio Imaging, Collégien, France), and further incubated for 1 h. Cells were then washed once with ice-cold phosphate-buffered saline (PBS) and lysed on ice for 30 min in lysis buffer containing 1% Triton X-100, 50 mM Tris-HCl (pH 7.4), 150 mM NaCl, 20 mM NaF, 2 mM Na_3_VO_4_, 2 mM PMSF, 0.5% glycerol and 0.1 mg mL^−1^ BSA. Lysates were scraped, sonicated (Vibra-Cell, Sonics & Materials, Inc., CT, USA) and cleared by centrifugation at 12,000× *g* for 15 min at 4 °C. Protein lysates were separated on 12% SDS-PAGE gels. After electrophoresis, proteins were transferred to polyvinylidene difluoride (PVDF) membranes using a wet-blot system. Membranes were blocked with 5% (*w*/*v*) bovine serum albumin (BSA) in Tris-buffered saline with Tween-20 (TBST) for 1 h at room temperature and incubated overnight at 4 °C with primary antibodies (all 1:1000): β-actin, p38, phospho-p38, c-Jun N-terminal kinase (JNK) (ABClonal, Wuhan, China) and phospho-JNK (Cell Signaling Technology, MA, USA). Following three TBST washes, membranes were probed with horseradish peroxidase (HRP)-conjugated rabbit IgG secondary antibody (Jackson ImmunoResearch Inc., West Grove, PA, USA) for 1 h at room temperature. Immunoreactive bands were visualized with NcmECL Ultra reagent (New Cell & Molecular Biotech Co., Ltd., Suzhou, China) and captured using a ChemiDoc™ XRS+ system with Image Lab™ 6.1 software (Bio-Rad, CA, USA). Band intensities were quantified with ImageJ 1.53e and normalized to the corresponding β-actin signal. Original figures can be found in Supplementary Materials.

### 2.5. Cell-Viability Assessment After 12 h Post-UVB

HaCaT cells were seeded in 96-well plates and allowed to attach overnight at 37 °C in 5% CO_2_. The medium was then replaced with serum-free DMEM, and cells were treated with graded concentrations of taxifolin, vehicle (ethanol), or the positive control gallic acid for 1 h. Subsequently, the plates were exposed to UVB irradiation (312 nm, 225 mJ cm^−2^) using the Bio-Sun system (Vilber Bio Imaging, Collégien, France); a non-irradiated group was included in parallel. After irradiation, cells were returned to the incubator for 12 h. Viability was determined by the MTT assay, and results are expressed as percentage survival relative to the untreated, non-irradiated control.

### 2.6. Assessment of Apoptosis (Hoechst 33258 Staining)

HaCaT cells were seeded in 96-well plates and allowed to attach overnight at 37 °C under 5% CO_2_. After replacing the medium with serum-free DMEM, cells were treated with graded concentrations of taxifolin or an equal volume of ethanol vehicle; a non-irradiated group was included in parallel, and incubation continued for 1 h. The plates were then exposed to UVB irradiation (312 nm, 225 mJ cm^−2^) using the Bio-Sun system (Vilber Bio Imaging, Collégien, France) and returned to the incubator for 12 h. Staining was performed according to the Beyotime Hoechst 33258 kit (Beyotime Biotechnology Co., Ltd., Shanghai, China): medium was aspirated, 200 μL fixative was added for 10 min, and wells were gently washed with PBS. Subsequently, 200 μL Hoechst 33258 working solution was applied for 5 min in the dark. After removing the dye, cells were washed again with PBS and kept in 50 μL PBS for imaging. Apoptotic morphology (nuclear condensation/fragmentation) was visualized and captured using an inverted fluorescence microscope (DMi8, Leica Microsystems, Wetzlar, Germany).

### 2.7. Dual-Fluorescence Viability/Cytotoxicity Assay (Calcein Acetoxymethyl Ester (Calcein-AM)/Propidium Iodide (PI) Co-Staining)

HaCaT cells were treated exactly as described for the Hoechst 33258 staining protocol (Section 2.6). Staining was performed according to the Beyotime Calcein-AM/PI kit (Beyotime Biotechnology Co., Ltd., Shanghai, China). Briefly, medium was aspirated, cells were gently washed with PBS, and 100 μL of Calcein-AM/PI working solution was added per well. After 30 min incubation at 37 °C in the dark, wells were washed twice with PBS and immediately observed under an inverted fluorescence microscope (DMi8, Leica Microsystems, Wetzlar, Germany ). Green fluorescence (Calcein-AM, Ex/Em 494/517 nm) indicates intact, viable cells; red fluorescence (PI, Ex/Em 535/617 nm) denotes membrane-compromised/dead cells.

### 2.8. Quantitative Flow-Cytometric Apoptosis Assay

HaCaT cells were seeded in Petri dishes and allowed to attach overnight at 37 °C under 5% CO_2_. After replacing the medium with serum-free DMEM, cells were treated with 100 μg mL^−1^ taxifolin or an equal volume of ethanol vehicle; a non-irradiated group was included in parallel, and incubation continued for 1 h. The dishes were then exposed to UVB irradiation (312 nm, 225 mJ cm^−2^) using the Bio-Sun system (Vilber Bio Imaging, Collégien, France) and returned to the incubator for 12 h. Apoptosis was quantified according to the Beyotime Annexin V-fluorescein isothiocyanate (FITC)/PI apoptosis detection kit (Beyotime Biotechnology Co., Ltd., Shanghai, China). Briefly, medium was aspirated, cells were gently washed with ice-cold PBS, and detached with 200 μL of 0.25% trypsin (EDTA-free) at 37 °C. The cell suspension was transferred to a 2 mL tube, diluted with PBS, and centrifuged at 1000× *g* for 5 min at 4 °C. After two washes with PBS, the pellet was resuspended in 195 μL of 1 × Binding Buffer. Five microliters of Annexin V-FITC and 10 μL of PI were added, mixed gently, and incubated for 15 min at room temperature in the dark. Samples were immediately analyzed on an ACEA NovoCyte flow cytometer (ACEA Biosciences, San Diego, CA, USA).

### 2.9. Transcriptomic Sequencing

HaCaT cells were seeded in Petri dishes and allowed to attach overnight at 37 °C under 5% CO_2_. After replacing the medium with serum-free DMEM, cells were treated with 100 μg mL^−1^ taxifolin or an equal volume of ethanol vehicle; a non-irradiated group was included in parallel, and incubation continued for 1 h. The dishes were then exposed to UVB irradiation (312 nm, 225 mJ cm^−2^) using the Bio-Sun system (Vilber Bio Imaging, Collégien, France) and returned to the incubator for 12 h. Culture medium was aspirated, cells were scraped on ice, and stored at −80 °C until shipment on dry ice to MajorBio Co., Ltd. (Shanghai, China) for RNA sequencing. Poly(A) mRNA was captured with oligo(dT)-coated magnetic beads, fragmented to ~300 nt, and reverse-transcribed into first-strand cDNA with random hexamers. After second-strand synthesis, adapters were ligated to both ends, and the libraries were size-selected, PCR-enriched, and purified. Final libraries were sequenced on the DNBSEQ-T7 platform (MGI Tech Co., Ltd., Shenzhen, China). Differential expression was analyzed by DESeq2; genes with |log_2_FC| ≥ 1, and adjusted *p*-value < 0.05 were considered significant. Functional enrichment (Gene Ontology (GO), Kyoto Encyclopedia of Genes and Genomes (KEGG), non-redundant protein database (NR)) and pathway analyses were performed to decipher the biological roles of the differentially expressed genes in the cellular response to UVB and taxifolin treatment. Transcriptomics data have been deposited in the NCBI BioProject database under accession PRJNA1405590.

### 2.10. Proteomic Analysis

HaCaT cells were treated exactly as described for the transcriptomic sequencing protocol (Section 2.9). HaCaT cells were harvested and stored at −80 °C until shipment on dry ice to MajorBio Co., Ltd. (Shanghai, China) for proteomic analysis. Data-independent acquisition (DIA) was performed on an Orbitrap Astral high-resolution mass spectrometer (Thermo Fisher Scientific, Waltham, MA, USA). Raw files were processed against the UniProt human database. Differential abundance was calculated with |log_2_FC| ≥ 1, and adjusted *p*-value < 0.05 as significance thresholds. Functional annotation and enrichment (GO, KEGG, NR) were conducted to delineate the biological roles of the differentially expressed proteins in the cellular response to UVB and taxifolin treatment. Mass spectrometry–based proteomics data have been deposited in the ProteomeXchange Consortium via the PRIDE partner repository [20] under accession PXD073279.

### 2.11. High-Performance Liquid Chromatography (HPLC) Assay of Taxifolin

Taxifolin was analyzed on a Shimadzu LC-20AR HPLC system (Kyoto, Japan) equipped with an SPD-M20A diode-array detector. Separation was achieved on a Shimadzu C18 column (5 µm, 4.6 mm × 150 mm) maintained at 30 °C. The mobile phase consisted of methanol and 0.1% acetic acid (60:40, *v*/*v*), delivered at 1.0 mL min^−1^. The 10 min run was monitored with full-spectrum ultraviolet-visible (UV-Vis) detection.

### 2.12. Statistical Analysis

Data are presented as mean ± standard deviation (SD). Inter-group differences were evaluated by one-way ANOVA followed by Duncan’s multiple-range test, with a significance threshold set at *p* < 0.05. All statistical analyses were performed using IBM SPSS Statistics v31 (IBM Corp., Armonk, NY, USA).

## 3. Results

### 3.1. Antioxidant Profile of Taxifolin

**Figure 1:**
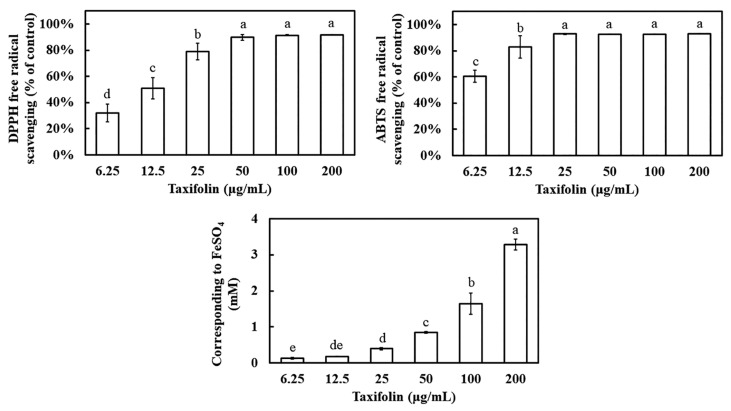
Concentration-dependent antioxidant activity of taxifolin assessed via DPPH and ABTS free radical scavenging assays, and ferric reducing antioxidant power (FRAP). DPPH and ABTS scavenging activities expressed as percentage of control, with activity increasing from 6.25 to 200 μg mL^−1^ taxifolin. Reducing capacity of FRAP was presented as FeSO_4_-equivalent concentration (mM). Different lowercase letters denote statistically significant differences between groups. Data are shown as mean ± standard deviation (error bars represent SD).

Both DPPH and ABTS assays revealed potent, concentration-dependent radical-scavenging activity (Figure 1). In the DPPH assay, taxifolin achieved 50% scavenging at 12.5 μg mL^−1^ and exceeded 90% removal at ≥50 μg mL^−1^, matching the performance of ascorbic acid (≥25 μg mL^−1^ for 90% scavenging) (Figure S1). The ABTS test was even more responsive: 6.25 μg mL^−1^ taxifolin produced 60.5% scavenging, equivalent to ascorbic acid at the same concentration, and the entire dose–response curve was shifted leftward, indicating higher affinity for ABTS than for DPPH. FRAP analysis further demonstrated a progressive increase in reducing power; at 200 μg mL^−1^, taxifolin generated a Fe^2+^ equivalent of 3.3 mM FeSO_4_, underscoring its robust electron-donating capacity.

### 3.2. ROS-Scavenging Efficacy of Taxifolin in HaCaT Cells

**Figure 2:**
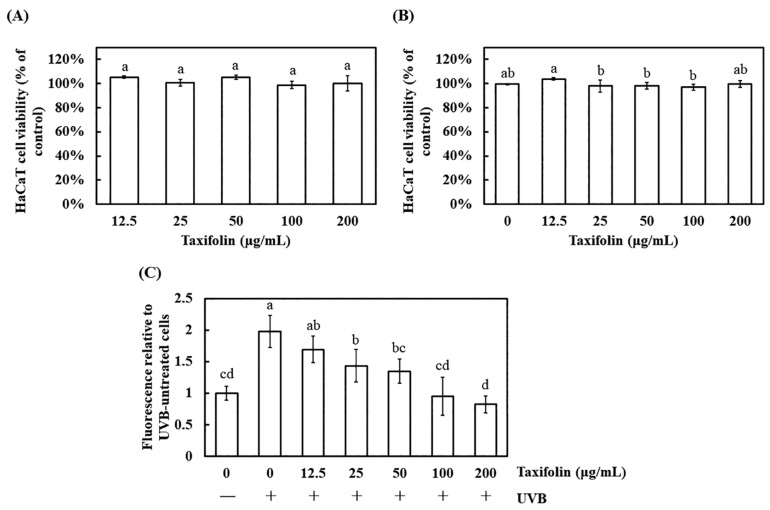
Effects of taxifolin on HaCaT cell viability and UVB-induced ROS generation. (**A**) Cell viability after 1 h exposure to taxifolin (12.5–200 μg mL^−1^) was quantified by MTT assay and expressed relative to the untreated control. (**B**) Cells were pre-incubated with taxifolin (0–200 μg mL^−1^) for 1 h, exposed to UVB (312 nm, 225 mJ cm^−2^) and assessed 1 h later by MTT assay. Cell viability is shown as a percentage of the non-irradiated, vehicle-only control. (**C**) Intracellular ROS levels were measured after 1 h post-irradiation by fluorescence intensity and normalized to the non-irradiated, vehicle-only control. Different lowercase letters denote statistically significant differences between groups. Data are shown as mean ± standard deviation (error bars represent SD).

Cell-compatibility was first verified by MTT. After 1 h exposure to 12.5–200 μg mL^−1^ taxifolin, viability remained >98%; the same range applied 1 h post-UVB (225 mJ cm^−2^) still yielded survivals ≥97%, indistinguishable from vehicle (Figure 2A,B). Thus, taxifolin exhibited no cytotoxicity within the short exposure window and did not interfere with acute UVB stress, validating its use for ROS assays.

Intracellular ROS were quantified with DCFH-DA. UVB elevated fluorescence 2-fold relative to the unirradiated control, confirming oxidative stress. Pre-treatment with taxifolin for 1 h dose-dependently quenched this burst; at 100 μg mL^−1^, signal returned to baseline with no statistical difference versus the non-UVB group, demonstrating complete suppression of UVB-induced ROS generation (Figure 2C).

### 3.3. Western Blot Analysis of JNK/p38 Phosphorylation

**Figure 3:**
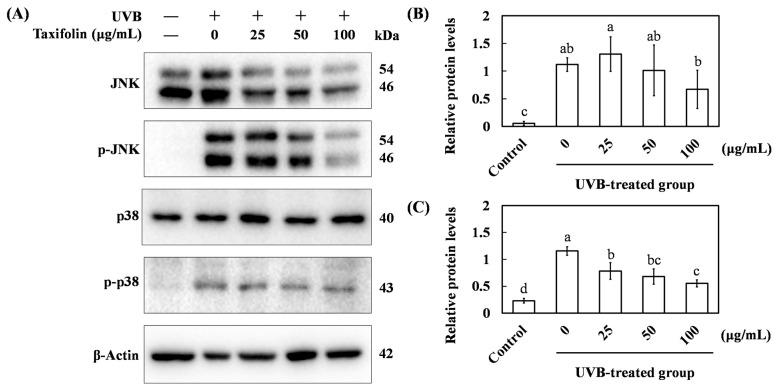
Taxifolin suppressed UVB-induced MAPK signaling in HaCaT keratinocytes. (**A**) Representative Western blots of total and phosphorylated JNK and p38 harvested 1 h after UVB irradiation (312 nm, 225 mJ cm^−2^) with or without taxifolin pre-treatment (0–100 µg mL^−1^). β-Actin served as a loading control. Relative band intensities (phospho/total) of JNK (**B**) and p38 (**C**) were quantified by ImageJ. Different lowercase letters denote statistically significant differences between groups. Data are shown as mean ± standard deviation (error bars represent SD).

UVB radiation activates the JNK/p38 MAPK cascade, amplifying ROS production and steering cells toward apoptosis. We therefore examined the effect of taxifolin on p38 and JNK phosphorylation. After 1 h exposure to UVB (225 mJ cm^−2^), p-p38 and p-JNK levels in HaCaT cells rose to 115.5% and 111.7% of control, respectively, confirming robust MAPK activation (Figure 3). Pre-incubation with taxifolin for 1 h suppressed this increase in a concentration-dependent manner; at 100 μg mL^−1^, p-p38 and p-JNK signals fell to 55.3% and 66.9%, respectively. These data indicate that taxifolin interrupted the ROS-MAPK by preventing the phosphorylation of p38 and JNK.

### 3.4. Evaluation of HaCaT Cell Viability After 12 h Post-UVB

**Figure 4:**
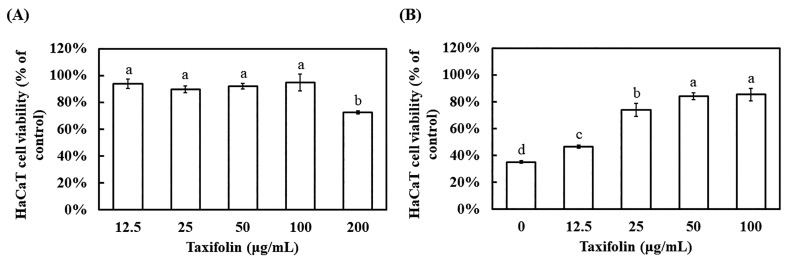
Effects of taxifolin on HaCaT cell viability. (**A**) Cell viability after 12 h exposure to taxifolin (12.5–200 μg mL^−1^) was quantified by MTT assay and expressed relative to the untreated control. (**B**) Cells were pre-incubated with taxifolin (0–100 μg mL^−1^) for 1 h, exposed to UVB (312 nm, 225 mJ cm^−2^) and assessed 12 h later by MTT assay. Cell viability is shown as a percentage of the non-irradiated, vehicle-only control. Different lowercase letters denote statistically significant differences between groups. Data are shown as mean ± standard deviation (error bars represent SD).

Extending the observation to 12 h allowed assessment of taxifolin’s ability to counteract delayed UVB injury. In the absence of irradiation, taxifolin ≤ 100 μg mL^−1^ did not affect basal viability, whereas 200 μg mL^−1^ reduced survival to 72.6%, indicating mild cytotoxicity upon prolonged high-dose exposure (Figure 4A). Twelve hours after UVB (225 mJ cm^−2^), vehicle-treated controls dropped to 35.2% viability (Figure 4B). Pre-incubation with taxifolin for 1 h dose-dependently reversed this loss: 50 μg mL^−1^ restored viability to 84.2%, equivalent to the positive control gallic acid (40 μg mL^−1^, 84.4%) (Figure S2). These data demonstrate that taxifolin effectively suppressed UVB-elicited delayed cell death and exhibited protective potency comparable to classical phenolic antioxidants.

**Figure 5:**
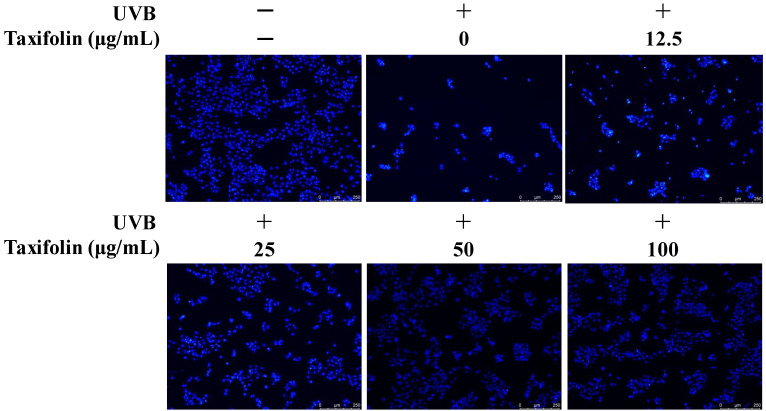
Taxifolin attenuated UVB-induced cell apoptosis. Cells were pre-treated with taxifolin (0–100 µg mL^−1^) for 1 h, exposed to UVB (312 nm, 225 mJ cm^−2^), and stained with Hoechst 33258 12 h later. Representative fluorescence micrographs illustrate a normal and an apoptotic cell.

Hoechst 33258 staining revealed that non-irradiated nuclei displayed uniform, blue fluorescence with intact contours (Figure 5). Twelve hours after UVB (225 mJ cm^−2^), the number of normal nuclei dropped sharply, and infrequent, intensely white fluorescent clumps appeared, indicative of chromatin condensation and fragmentation, hallmarks of apoptotic nuclear damage. Pretreatment with taxifolin produced a clear dose–response: at 12.5 μg mL^−1,^ the proportion of intact nuclei increased slightly, yet many bright, condensed bodies remained. When the concentration reached 50–100 μg mL^−1^, uniform blue nuclei predominated and brightly stained, fragmented nuclei were almost absent. These observations confirm that taxifolin effectively suppresses UVB-induced nuclear injury and prevents cells from entering the apoptotic program.

**Figure 6:**
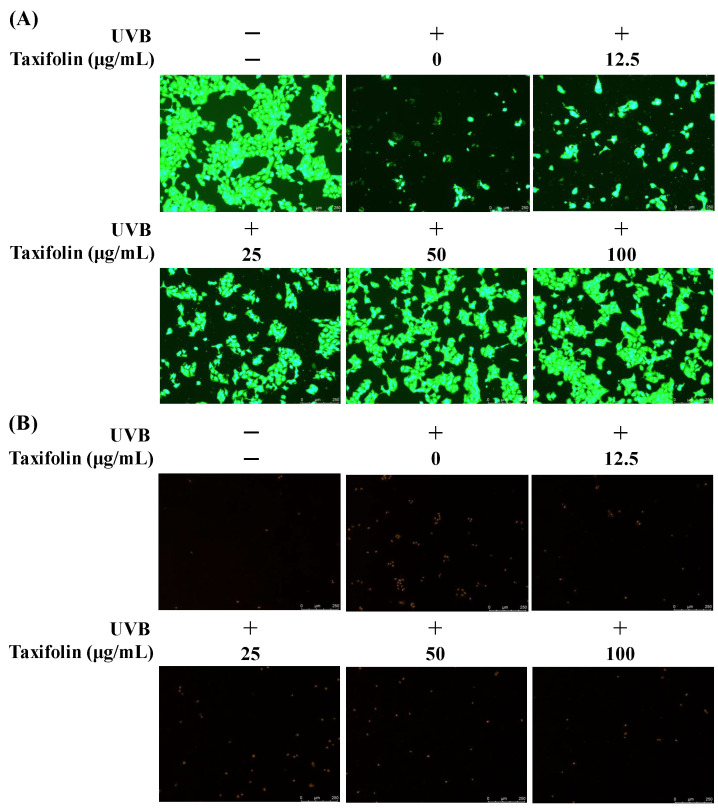
Calcein-AM/PI co-staining of HaCaT cells after 12 h post-UVB treatment. Cells were pre-treated with taxifolin (0–100 µg mL^−1^) for 1 h, irradiated (312 nm, 225 mJ cm^−2^), and stained with Calcein-AM ((**A**); green, live cells) and propidium iodide ((**B**); red, dead cells). Representative fluorescence micrographs show dose-dependent preservation of plasma-membrane integrity and reduction in UVB-induced cytotoxicity.

Calcein-AM/PI dual staining corroborated the nuclear morphology data. In non-irradiated controls, dense green fluorescence dominated and red signals were minimal (Figure 6). Twelve hours after UVB (225 mJ cm^−2^), green intensity declined markedly and red fluorescence became abundant, reflecting loss of membrane integrity and reduced viability. Pretreatment with taxifolin reversed this trend in a concentration-dependent manner. Green fluorescence gradually recovered and red signals diminished as the drug dose increased. At 100 μg mL^−1^, PI-positive cells were markedly reduced and the proportion of viable cells rose substantially. Collectively, taxifolin effectively preserved membrane integrity and significantly suppressed UVB-induced cell death.

**Figure 7:**
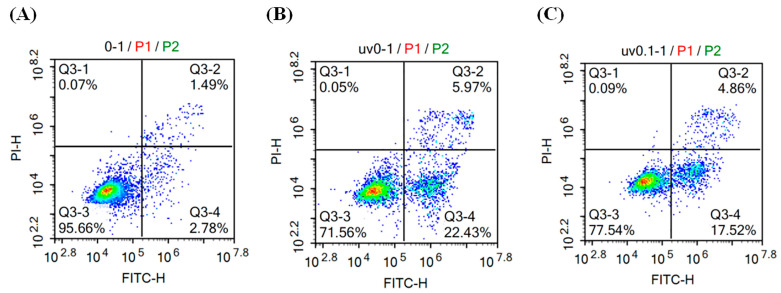
Flow cytometric quantification of apoptosis in HaCaT cells after 12 h post-UVB treatment (312 nm, 225 mJ cm^−2^). Cells were stained with Annexin V-FITC/PI and analyzed (**A**) without UVB, (**B**) with UVB alone, and (**C**) with UVB followed by taxifolin (100 µg mL^−1^) treatment. Representative flow-cytometry dot plots are shown.

Flow-cytometric quantification (Annexin V-FITC/PI) corroborated the morphological findings. In non-irradiated controls, 95.77 ± 0.18% of cells were viable (Q3-3), with total apoptosis (Q3-2 + Q3-4) at only 4.13 ± 0.17% and necrosis (Q3-1) as low as 0.10 ± 0.04% (Figure 7). Twelve hours after UVB (225 mJ cm^−2^), viability dropped sharply to 71.61 ± 0.36%, whereas total apoptosis rose significantly to 28.30 ± 0.39%, indicating that UVB predominantly triggered apoptotic cell death. Pretreatment with 100 μg mL^−1^ taxifolin for 1 h before irradiation restored viability to 77.15 ± 0.51% and reduced total apoptosis to 22.74 ± 0.54%. While the flow-cytometric analysis of cell viability showed a difference from the MTT results, this likely reflects their measurement of different biological endpoints: mitochondrial metabolic activity in the MTT assay versus early apoptotic membrane changes detected by Annexin V staining. These data further confirm that taxifolin effectively suppressed UVB-induced apoptotic signaling and improved long-term cell survival.

### 3.5. Multi-Omics Dissection of Taxifolin’s Molecular Intervention Network Under UVB Stress

To delineate the baseline UVB signature, we compared the UV_C group (UVB-irradiated vehicle control) with the Ctrl group (non-irradiated control). At the transcriptome level, 6489 genes were significantly altered (2760 up- and 3729 down-regulated), while proteomics identified 1875 differential proteins (560 up- and 1315 down-regulated), underscoring the genome-wide transcriptional–translational perturbation imposed by UVB (Figure S3). We contrasted the Tax group (UVB + 100 μg mL^−1^ taxifolin) with the UV_C group. Taxifolin intervention yielded 605 differential transcripts (585 up, 20 down) and 379 differential proteins (218 up, 161 down), indicating a pronounced reversal of UVB-driven expression imbalance. Hierarchical clustering revealed that the Tax samples formed a discrete branch, separate from the UV_C cluster, at both the RNA and protein levels, confirming that taxifolin remodels the UVB-induced molecular signature (Figure S4).

**Figure 8:**
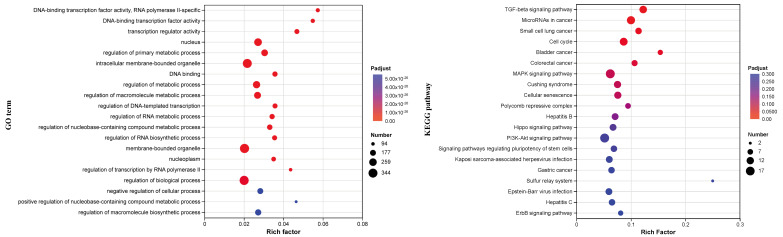
Functional assay of taxifolin-regulated transcripts in UVB-injured HaCaT cells. GO and KEGG pathway enrichment analyses of differentially expressed genes between taxifolin (100 µg mL^−1^) and vehicle groups after 12 h post-UVB treatment (312 nm, 225 mJ cm^−2^).

**Figure 9:**
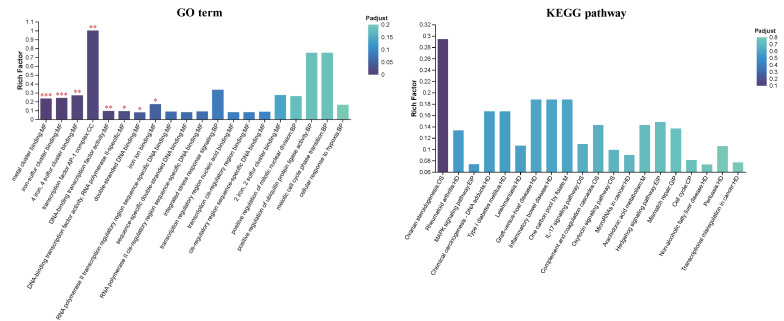
Functional assay of taxifolin-regulated proteins in UVB-injured HaCaT cells. GO and KEGG pathway enrichment analyses of differentially expressed proteins between taxifolin (100 µg mL^−1^) and vehicle groups after 12 h post-UVB treatment (312 nm, 225 mJ cm^−2^). ***, **, and * indicate statistically significant differences at p-adjusted < 0.001, < 0.01, and < 0.05, respectively.

**Figure 10:**
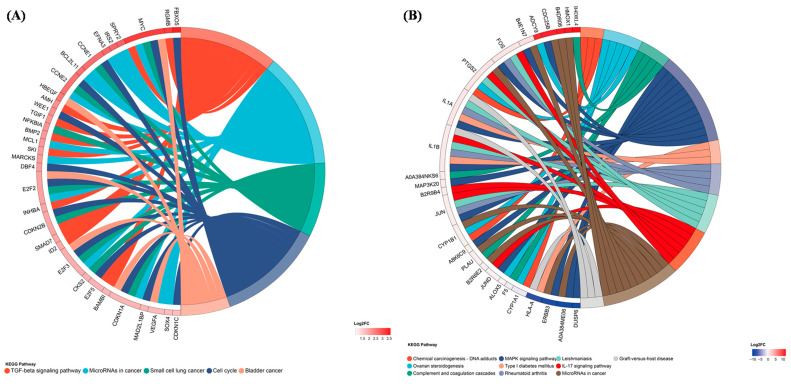
KEGG enrichment chord networks linking taxifolin-modulated pathways to representative core genes/proteins in UVB-injured HaCaT cells. Outer arcs represent significantly enriched KEGG terms; inner ribbons connect each pathway to its differentially (**A**) transcripts and (**B**) proteins identified between taxifolin (100 µg mL^−1^) and vehicle groups after 12 h post-UVB treatment (312 nm, 225 mJ cm^−2^). CCNE1, CCNE2, HMOX1, B4DR06, and B4E1N7 indicate cyclin E1, cyclin E2, heme oxygenase-1, cyclin D1, and Myc proto-oncogene protein.

Gene-set enrichment against the GO database revealed that UVB-responsive transcripts were markedly over-represented in DNA-binding transcription factor activity, nucleus, membrane-bounded organelle and regulation of biological/metabolic processes. KEGG analysis further highlighted oncogenic routes (small-cell lung, bladder and colorectal cancer) together with signaling cascades such as TGF-β, MAPK and cell-cycle, as well as cellular senescence and virus-infection pathways (Figure 8). At the proteome level, GO terms were dominated by molecular-function categories including metal-cluster binding, iron–sulfur cluster binding and DNA-binding transcription factor activity (Figure 9). Although individual KEGG terms did not reach overwhelming significance, the mapped proteins clustered around cancer-related modules (ovarian steroidogenesis, chemical carcinogenesis–DNA adducts) and the MAPK signaling axis. Chord plots constructed for each enriched term illustrate the direction of change. Following taxifolin intervention, the majority of differential transcripts were up-regulated (e.g., MYC, CCNE1 (cyclin E1), E2F2, E2F3, E2F5, SKI) and converged on cell-cycle, TGF-β and cancer-associated pathways (Figure 10). Likewise, most proteins showed increased abundance (e.g., HMOX1 (heme oxygenase-1), JUN, JUND, FOS, B4E1N7 (Myc proto-oncogene protein), B4DR06 (cyclin D1)), whereas a smaller subset was down-regulated and linked to microRNAs in cancer and the MAPK signaling pathway. Together, these patterns indicate that taxifolin reprogrammed UVB-driven transcriptional and translational landscapes toward survival and repair rather than malignant progression.

**Figure 11:**
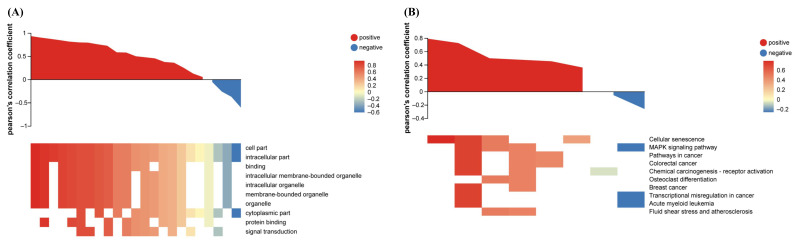
Associated functional assay of taxifolin-regulated transcripts and proteins in UVB-injured HaCaT cells. (**A**) GO and (**B**) KEGG pathway enrichment analyses of differentially expressed genes and proteins between taxifolin (100 µg mL^−1^) and vehicle groups after 12 h post-UVB treatment (312 nm, 225 mJ cm^−2^).

Pearson correlation of transcript abundance versus protein abundance yielded r = 0.35858, indicating modest overall correspondence—an expected divergence attributable to post-transcriptional, translational and post-translational regulation (Figure S5). Nevertheless, when GO terms and KEGG pathways with significant representation in both datasets were examined, a strong positive correlation emerged, implying concordant directional change at the functional level (Figure 11). Focusing on molecules that met the stringent “transcript–protein association” criterion (|log_2_FC| ≥ 1 in both layers), taxifolin intervention produced highly coherent up-regulation (Table 1). Fourteen genes were concurrently up-regulated at both the transcriptional and protein levels, whereas only one gene showed down-regulation. These core targets converge on cell proliferation, stress adaptation, and cell cycle regulation, collectively underpinning the protective program against UVB injury in HaCaT keratinocytes.

## 4. Discussion

Previous studies have established that taxifolin possesses radical-scavenging capacity comparable to that of Trolox [7,8]. In the present study, we extended the comparison to vitamin C (ascorbic acid) and found that taxifolin’s antioxidant activity was comparable (Figure 1). In HaCaT cells challenged with UVB, irradiation sharply elevated intracellular ROS, whereas taxifolin restored basal levels (Figure 2), consistent with its ROS-suppressive effect reported in melanoma models [9,10]. Mechanistically, UVB rapidly triggered phosphorylation of JNK and p38, up-regulated pro-apoptotic genes and amplified the inflammatory/ROS feed-forward loop that drives cells toward apoptosis and malignant transformation [21]. Taxifolin markedly attenuated activation of both stress-activated MAPK branches (Figure 3). This finding is consistent with observations from unrelated stress paradigms. In pulmonary embolism and arterial thrombosis models, taxifolin stabilized thrombus formation by curbing phosphorylation of key MAPK and PI3K/Akt axis proteins (ERK, p38, JNK, Akt) [15]. In LPS-stimulated RAW264.7 macrophages, taxifolin-mediated blockade of JNK and p38 hyperphosphorylation was statistically indistinguishable from that of benchmark inhibitors SP600125 and SB203580, conferring potent anti-inflammatory activity [22]. Collectively, these data demonstrate that taxifolin consistently disrupted the ROS–MAPK vicious cycle across multiple experimental settings.

Moreover, published UV spectra report taxifolin’s absorption maximum at approximately 290 nm, with negligible absorbance beyond 400 nm [23]. Our HPLC chromatogram reproduced this profile, yielding a single intense peak at 289 nm (Figure S6). Because the UVB spans 280–320 nm, taxifolin’s absorption maximum coincides with the most energetic portion of UVB, indicating that—beyond its antioxidant and signaling actions—taxifolin can act as a chemical sunscreen, intercepting photons before they reach cellular targets and thereby reinforcing its overall photoprotective capacity.

Taken together, MTT viability (Figure 4), fluorescence imaging (Figure 5 and Figure 6) and flow-cytometric assays (Figure 7) showed that taxifolin sharply attenuated UVB-induced apoptosis in HaCaT keratinocytes and restored overall survival. This anti-apoptotic efficacy mirrors observations across diverse pathological settings. In hepatic ischemia–reperfusion injury, taxifolin bolstered PINK1/Parkin-mediated mitophagy, curbed ROS burst and suppressed hepatocyte apoptosis [11]. In glucocorticoid-induced osteonecrosis, it activated Nrf2, inhibited NADPH oxidase, reduced osteoblast apoptosis and improved trabecular micro-architecture [13]. Furthermore, in terms of cardiovascular protection, multiple studies have also demonstrated that taxifolin can reduce the apoptosis of cardiomyocytes [6,14]. These cross-model data converge on a unified theme: through a multi-target antioxidant network, taxifolin efficiently aborts apoptotic programs triggered by exogenous toxins or oxidative stress.

To dissect the photoprotective mechanism of taxifolin in an unbiased manner, we performed parallel transcriptomic and proteomic profiling and visualized the data as an enrichment chord network that couples statistical significance with fold-change magnitude. This integrative map immediately reveals which functional modules are reprogrammed and which driver genes/proteins execute the switch (Figure 10). Intersection analysis identified the G1/S transition of the cell cycle as the pathway simultaneously enriched and most prominently altered at both the RNA and protein levels. The core engines of this transition, CCNE1 (Cyclin E1) and CCNE2 (Cyclin E2), associate with CDK2 to hyper-phosphorylate and inactive the retinoblastoma protein (Rb), thereby liberating E2 promoter-binding factor (E2F) transcription factors and launching the S-phase gene-expression program [24]. Importantly, high Cyclin-E output is a well-established accelerator of neoplastic growth. In non-small-cell lung cancer, bone marrow mesenchymal stem cell (BMMSC)-derived exosomal miR-144 restrains tumor progression by down-regulating CCNE1/2 [25], whereas in bladder cancer, the transcription factor motor neuron and pancreas homeobox 1 (MNX1) binds directly to the CCNE1/2 promoters and enhances malignant proliferation [26]. Likewise, Cyclin D1, which promotes G1/S transition by phosphorylating Rb through CDK4/6, is frequently overexpressed when the cycle becomes oncogenically uncoupled [27]. Taxifolin did not provoke such carcinogenic overexpression. Instead, potentially reflecting reduced UVB-induced DNA damage burden, it restored the physiological abundance of Cyclin E and Cyclin D1 that UVB had suppressed, thereby alleviating the G1/S blockade. The net effect may be a timely exit from the cell cycle once repair is complete, lowering the likelihood of apoptosis.

Moreover, the proto-oncogene MYC (c-Myc) operates as a master switch of the cell cycle. It simultaneously up-regulates Cyclins, CDKs and E2Fs while repressing the CDK inhibitors CDKN1A/B, thereby driving cells across the G1/S boundary [28,29]. Down-regulation of MYC has been shown to remodel the tumor immune micro-environment (TIME) and sensitize tumors to immunotherapy, whereas its up-regulation is a potent mitogenic cue. In chronic rhinosinusitis with nasal polyps (CRSwNP), for example, c-Myc levels rise in direct proportion to CYR61-driven proliferation, paralleling an increase in cyclin D1 (CCND1) [30]. As a transcription factor, c-Myc precisely orchestrates proliferation, differentiation and metabolism. Integration of our transcriptomic and proteomic datasets in the KEGG enrichment chord network reveals that taxifolin modulated MYC expression, thereby curbing cycle dysregulation. By restoring physiological MYC levels, taxifolin re-establishes orderly proliferation and ultimately boosts HaCaT survival after UVB insult.

In addition, the proteomic chord diagram identified Jun and c-Fos as the most significantly up-regulated proteins at both transcript and protein levels. Members of the Jun family (c-Jun, JunD) together with c-Fos constitute the core of activator protein-1 (AP-1), whose biological output is exquisitely context-dependent [31]. Accumulating evidence shows that, in a variety of models, c-Jun elevation is coupled to pro-survival rather than pro-death signaling [32]. In immortalized H9c2 rat embryonic cardiomyocytes, c-Jun negatively regulates pro-apoptotic proteins such as cleaved caspase-3, cleaved caspase-9, Bax, and Bim. It also represses PTEN (phosphorylated PTEN), attenuating oxidative-stress-induced death through the PTEN/Akt signaling pathway [32]. An analogous cytoprotective role has been documented in c-jun^−^/^−^ mouse embryonic fibroblasts rescued by c-Jun re-expression [33]. Likewise, JunD decreases mortality in UV/H_2_O_2_-challenged mouse embryonic fibroblast cells [34,35], mirroring the pro-survival signature we observe here.

Furthermore, c-Fos—the other cornerstone of AP-1—is an immediate-early gene whose up-regulation is a hallmark of growth-factor or oxidative-stress responses [36,37]. Like c-Jun, high c-Fos expression has repeatedly been linked to enhanced proliferation and survival. Several studies showed that c-Fos/JunD heterodimers potently suppress stress-induced death and demonstrate that elevated AP-1 activity can switch from pro-apoptotic to pro-survival depending on cellular context [34,38]. Mechanistically, c-Fos stabilizes nuclear Cyclin D1, up-regulates cell-cycle proteins and anti-apoptotic factors, thereby aborting the apoptotic cascade. In human hepatocytes, c-Fos overexpression drives cells into S-phase by securing nuclear Cyclin D1 and boosts proliferation rates [39]. In rat pheochromocytoma (PC12) cells, concurrent up-regulation of c-Fos and Bcl-2/Bcl-xL not only curbs hypoxia-triggered apoptosis but also elevates neural-plasticity-related proteins, offering dual protection for post-stroke recovery [40]. Our proteomic chord network shows that taxifolin coordinately induced both c-Fos and c-Jun, implying that the flavonoid reinforced an AP-1-dependent pro-survival transcriptional program. This action further fortified the anti-apoptotic shield of HaCaT keratinocytes under UVB stress, preserving the cell viability.

Taken together, it is important to distinguish physiological cell-cycle recovery from pathological proliferation in the context of UVB-induced genotoxic stress. In our study, the observed up-regulation of MYC, Cyclin E/D1, E2Fs, and AP-1 components, as revealed by integrated transcriptomic and proteomic profiling, was moderate and fell within a range consistent with a physiological recovery response, distinct from the massive overexpression typically seen in cancer cells [24,41]. Furthermore, Cyclin D1 possesses a direct role in DNA damage repair; it is recruited to damage sites to facilitate RAD51 loading and promote homologous recombination through a breast cancer 2 susceptibility protein (BRCA2)-dependent mechanism [42]. Secondly, the inhibition of JNK/p38 phosphorylation occurred at an early stage of oxidative stress (1 h post-UVB irradiation). This timing suggests it primarily interrupts the ROS-MAPK feedback loop, rather than interfering with the Ataxia-telangiectasia mutated (ATM)/Ataxia-telangiectasia and Rad3-related (ATR)-mediated DNA damage checkpoints. This moderate suppression of MAPK may create a favorable environment for DNA repair by mitigating oxidative damage. Finally, the consistent up-regulation of c-Jun and c-Fos activates an AP-1-dependent pro-survival transcriptional program, which has been demonstrated to suppress apoptosis and promote DNA damage repair in various stress models. Therefore, the overall expression patterns we observed likely reflect a state of DNA-damage response and cellular adaptation rather than checkpoint bypass.

Additionally, integrated transcript-protein profiling (Table 1) shows that MYC and FOS were concordantly up-regulated >2-fold at both the RNA and protein levels, corroborating the chord-diagram signature. A third transcription factor, FOXQ1 (Forkhead box Q1), showed the same up-regulation in response to taxifolin. Although FOXQ1 plays a key role in epithelial malignancies and is linked to invasion, metastasis and poor prognosis, its biological output is context-dependent [43]. In colorectal cancer (CRC) cells, FOXQ1 transcriptionally up-regulates sirtuin 1 (SIRT1), promotes p53 de-acetylation, and thereby short-circuits p53-driven apoptosis, reinforcing platinum resistance (DNA-damage response) [44]. Conversely, in osteoarthritic chondrocytes, FOXQ1 suppresses the NOD-like receptor family pyrin domain containing 3 (NLRP3), Caspase-1 and gasdermin D (GSDMD)—the core pyroptotic machinery—curtailing IL-6, IL-18 and TNF-α release and conferring joint protection [45]. Thus, within the UVB-stressed keratinocyte milieu, taxifolin-evoked FOXQ1 induction appeared to prioritize an anti-apoptotic rather than a pro-oncogenic program.

Concomitant with FOXQ1 up-regulation, HMOX1 (heme oxygenase-1), the archetypal stress-inducible enzyme that catabolizes heme into biliverdin, carbon monoxide, and ferrous ions, was also induced, thereby conferring antioxidant, anti-inflammatory, and anti-apoptotic benefits [46]. Its up-regulation scavenges ROS, relieves endoplasmic-reticulum stress, and, via the PI3K/Akt/mTOR axis, promotes cell survival [47,48]. Yet the role of HMOX1 is not static; its impact on cell fate is context-dependent and bidirectional. In several cancers, most notably ovarian cancer, high HMOX1 is a key regulator of prognosis and immune-cell infiltration that can amplify malignant traits [49]. Nevertheless, in the acute oxidative-damage paradigm created by UVB, the rapid surge of HMOX1 was overwhelmingly interpreted as a cytoprotective switch that secured keratinocyte survival.

HaCaT cells are immortalized with mutant p53, potentially altering UVB sensitivity and DNA damage responses, and the monolayer format lacks the three-dimensional architecture, melanin content, and immunological microenvironment of native skin. These features limit extrapolation to primary keratinocytes and in vivo conditions. Nevertheless, this model offers experimental consistency and reproducibility for mechanistic exploration. Additionally, the relatively high taxifolin concentration (100 μg/mL) required for optimal protection raises questions about physiological relevance given its limited bioavailability. However, taxifolin has demonstrated favorable safety profiles: an intraperitoneal LD50 of 1200 mg/kg in rats [50], and no definitive evidence of mutagenicity, carcinogenicity, or reproductive toxicity. Regarding delivery, oral bioavailability varies by species (0.49% in rats [51], vs. 36% in rabbits [52]), while topical application achieves 48.09% percutaneous penetration in human skin ex vivo [53], supporting its potential for UV-induced skin protection despite the high in vitro concentration required.

## 5. Conclusions

In conclusion, this study demonstrates that taxifolin confers robust photoprotection against UVB-induced injury in HaCaT keratinocytes by modulating a multi-pronged defense system. Taxifolin suppressed oxidative stress-driven apoptosis and restored physiological homeostasis. Matching ascorbic acid in antioxidant capacity, taxifolin additionally acted as a chemical sunscreen. Its absorption maximum (≈290 nm) overlapped with the UVB spectrum (280–320 nm), enabling it to intercept photons before they reach cellular DNA and suppress the initial ROS burst. This upstream intervention inhibited the downstream phosphorylation of JNK/p38 and severed the ROS-MAPK vicious cycle at its source. Integrative transcriptomic and proteomic profiling revealed that taxifolin was associated with attenuation of UVB-induced G1/S cell cycle arrest concomitant with restored expression of Cyclin E and Cyclin D1. Meanwhile, it up-regulated the expression of MYC, FOXQ1, and HMOX1, reinstating a physiological proliferation program associated with DNA-damage response and cytoprotection. Furthermore, the coordinated induction of c-Jun and c-Fos boosted AP-1 activity, which in turn drove expression of anti-apoptotic and cell-cycle-progression genes, thereby reinforcing keratinocyte viability under UVB stress. Collectively, taxifolin integrated ROS-MAPK neutralization, cell cycle normalization, and AP-1-mediated survival signaling into a cohesive photoprotective network. This finding not only provides a compelling mechanistic framework for advancing taxifolin as a natural, multi-target photoprotectant but also identifies new molecular nodes for therapeutic intervention in UV-driven skin disorders.

## Figures and Tables

**Table 1:** Differentially expressed genes and proteins (log_2_FC ≥ 1) comparing taxifolin (100 µg mL^−1^) and vehicle groups after 12 h post-UVB treatment (312 nm, 225 mJ cm^−2^).

Regulation	Gene	Gene Description	Transcriptome	Proteome
			log_2_FC	
Up	TRIB1	Tribbles pseudokinase 1	3.765	11.290
	MYC	Myc proto-oncogene protein	3.167	1.295
	BAG36089.1	Creatine kinase M-type	2.968	1.566
	HBB	Hemoglobin subunit beta	2.849	1.218
	PKLR	Pyruvate kinase L/R	2.232	12.600
	A0A0F6QNT9	RING-type E3 ubiquitin transferase	1.649	3.350
	FOS	Protein c-Fos	1.523	1.08
	ATF3	Cyclic AMP-dependent transcription factor	1.521	1.655
	PMAIP1	Phorbol-12-myristate-13-acetate-induced protein 1	1.398	1.454
	HERPUD1	Homocysteine-inducible endoplasmic reticulum stress-inducible ubiquitin-like domain member 1	1.370	10.24
	BAG51270.1	mRNA decay activator protein ZFP36	1.277	1.072
	HMOX1	Heme oxygenase 1	1.199	11.440
	FOXQ1	Forkhead box protein Q1	1.025	10.330
	IRX2	Iroquois-class homeodomain protein IRX-2	1.006	1.035
Down	CST5	Cystatin-D	−2.500	−2.036

## Data Availability

The original contributions presented in this study are included in the article/Supplementary Material. Further inquiries can be directed to the corresponding author(s).

## Supplementary Materials

The following supporting information can be downloaded at: https://www.mdpi.com/article/10.3390/biom16030387/s1, Figure S1: Concentration-dependent antioxidant activity of ascorbic acid assessed via DPPH and ABTS free radical scavenging assays; Figure S2: Effects of gallic acid on HaCaT cell viability; Figure S3: Overview of differential expression profiles in UVB-injured HaCaT cells; Figure S4: Heatmaps of differential expression profiles in UVB-injured HaCaT cells; Figure S5: Following Venn-based intersection of the proteome and transcriptome datasets, a protein–transcript correlation was constructed for the resultant consensus gene set; Figure S6: Full-spectrum HPLC analysis of taxifolin.

## Abbreviations

The following abbreviations are used in this manuscript:

MTT	3-(4,5-Dimethylthiazol-2-yl)-2,5-diphenyltetrazolium bromide
ROS	Reactive oxygen species
JNK	c-Jun N-terminal kinase
MAPK	Mitogen-activated protein kinase
MYC	Myc proto-oncogene protein
FOXQ1	Forkhead box protein Q1
HMOX1	Heme oxygenase 1
AP-1	Activator protein-1
DPPH	2,2-Diphenyl-1-picrylhydrazyl
ABTS	2,2′-Azinobis-(3-ethylbenzothiazoline-6-sulfonic acid)
FRAP	Ferric reducing antioxidant power
CCNE 1	Cyclin E1
CCNE 2	Cyclin E2
CCND1	Cyclin D1
