# Supplementary material for: Multi-Target Photoprotection by Taxifolin Against UVB-Induced Keratinocyte Injury Through UVB Filtration, ROS Scavenging and Transcriptomic-Proteomic Reprogramming
Source: Biomolecules. 2026 Mar 4;16(3):387. doi: 10.3390/biom16030387 (PMC13024054; doi:10.3390/biom16030387)
Supplement: Supplementary file 1 [file biomolecules-16-00387-s001.zip › biomolecules-4150197-supplementary .pdf]

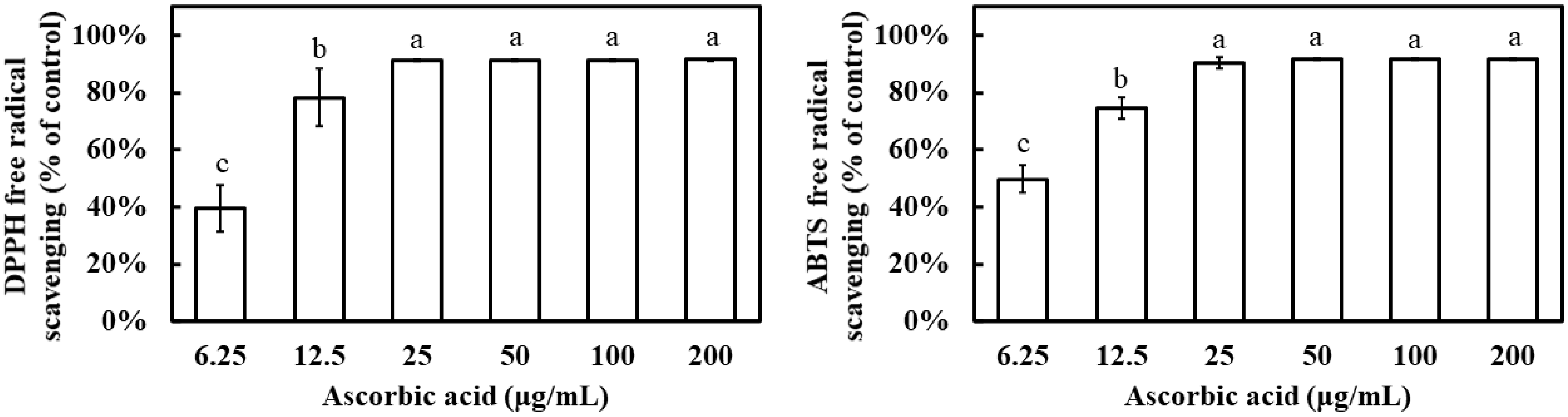

Figure S1. Concentration-dependent antioxidant activity of ascorbic acid assessed via DPPH and ABTS free radical scavenging assays. DPPH and ABTS scavenging activities expressed as percentage of control, with activity increasing from 6.25 to 200 μg mL^{-1} ascorbic acid. Different lowercase letters denote statistically significant differences between group. Data are shown as mean ± standard deviation (error bars represent SD).

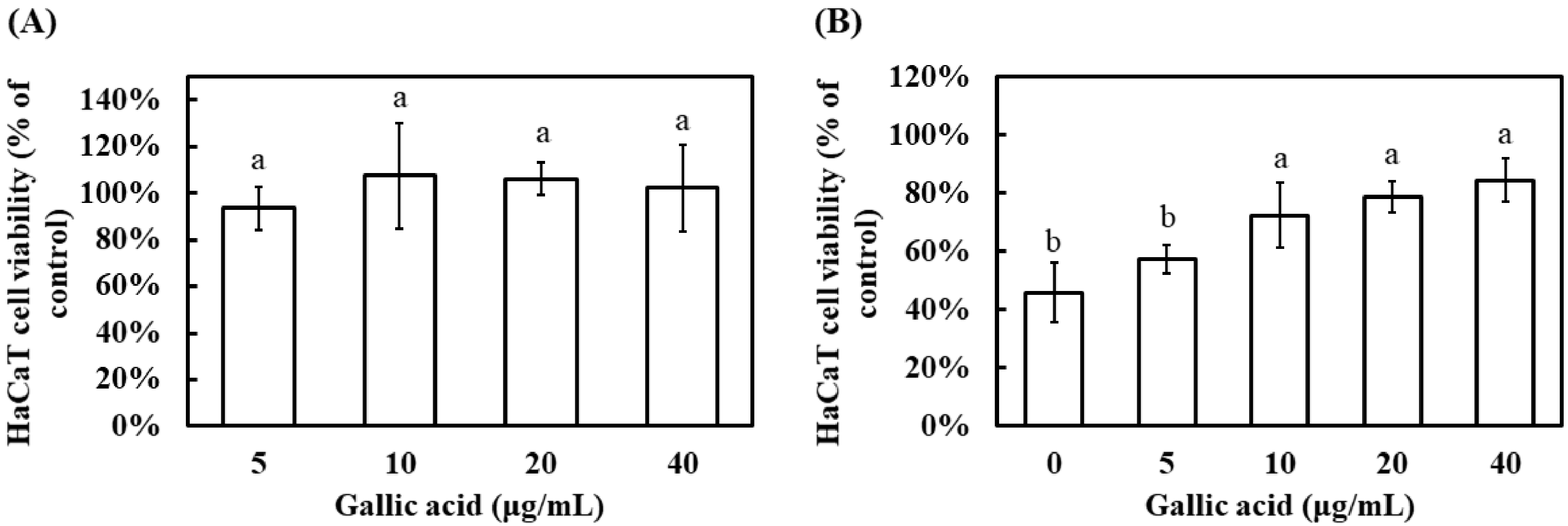

Figure S2. Effects of gallic acid on HaCaT cell viability. (A) Cell viability after 12 h exposure to taxifolin (5–40 μg mL^{-1}) was quantified by MTT assay and expressed relative to the untreated control. (B) Cells were pre-incubated with taxifolin (5–40 μg mL^{-1}) for 1 h, exposed to UVB (312 nm, 225 mJ cm^{-2}) and assessed 12 h later by MTT assay. Cell viability is shown as a percentage of the non-irradiated, vehicle-only control. Different lowercase letters denote statistically significant differences between group. Data are shown as mean ± standard deviation (error bars represent SD).

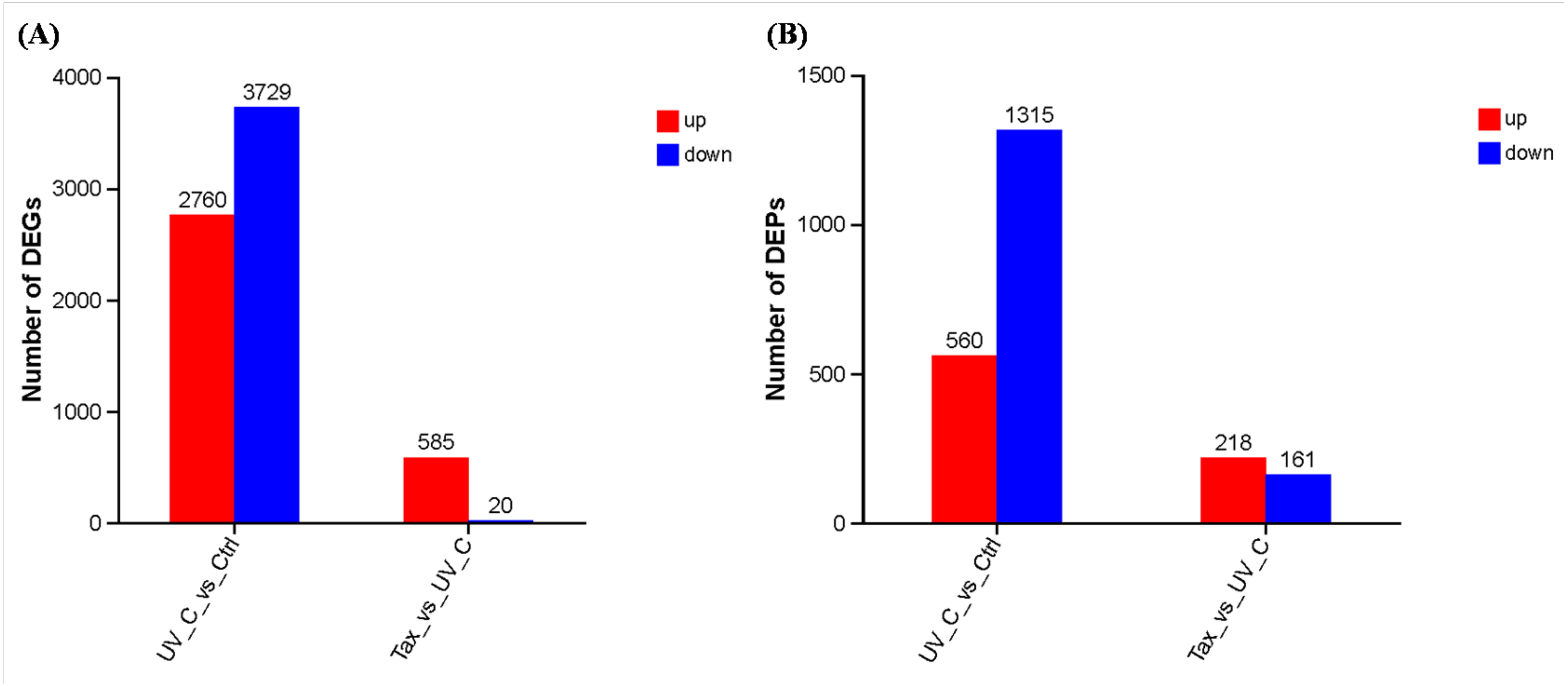

Figure S3. Overview of differential expression profiles in UVB-injured HaCaT cells. (A) Transcriptome and (B) proteome assays comparing taxifolin-treated (Tax, 100 μg mL^{-1}) versus vehicle-only (UV_C) groups after 12 h post-UVB treatment (312 nm, 225 mJ cm^{-2}). Ctrl denotes non-irradiated, vehicle-only control. Red and blue bar charts indicate significantly up- or down-regulated entities, respectively.

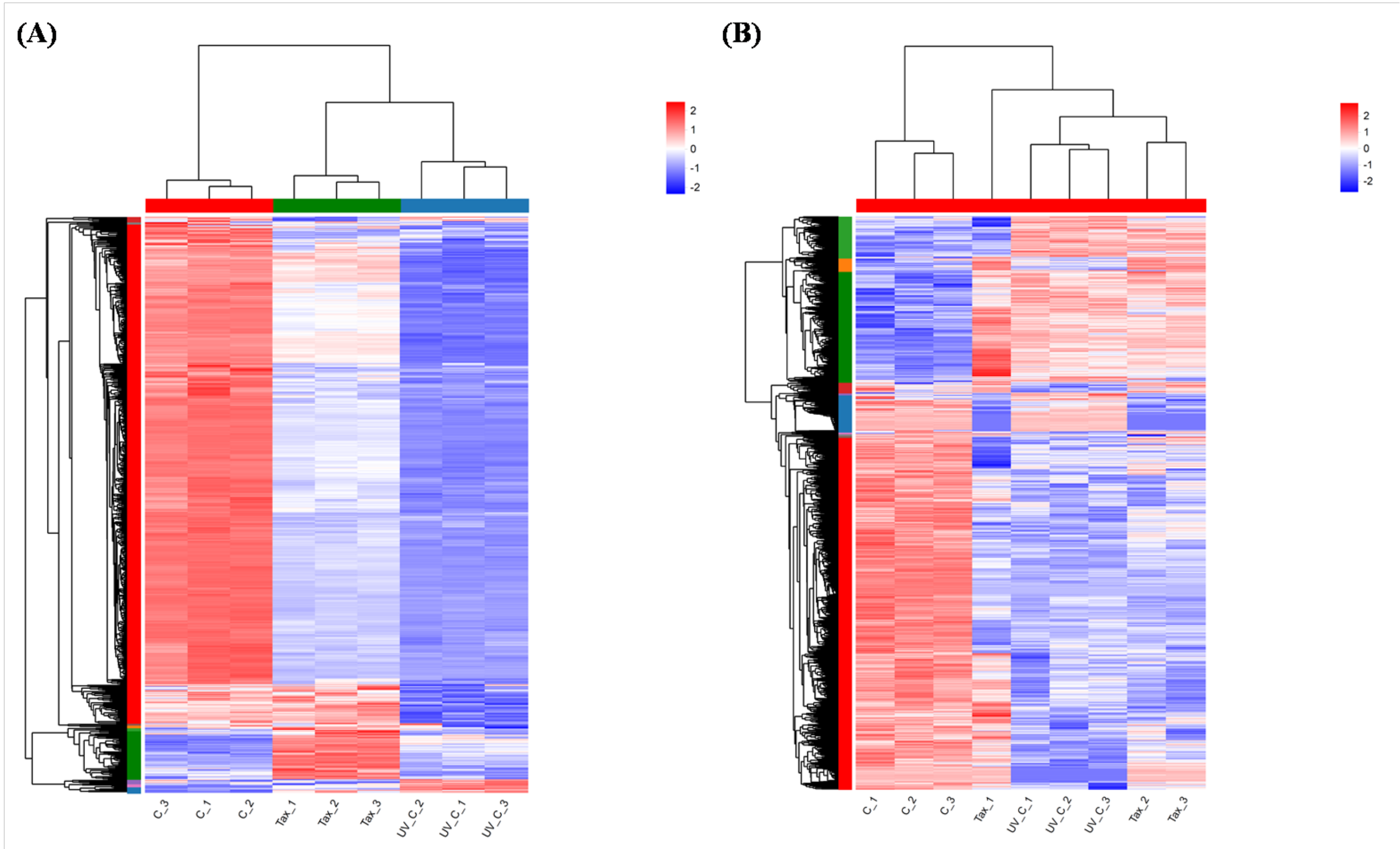

Figure S4. Heatmaps of differential expression profiles in UVB-injured HaCaT cells. (A) Transcriptome and (B) proteome assays comparing taxifolin-treated (Tax, 100 μg mL^{-1}) versus vehicle-only (UV_C) groups after 12 h post-UVB treatment (312 nm, 225 mJ cm^{-2}). Ctrl denotes non-irradiated, vehicle-only control. Data are shown for three biological replicates.

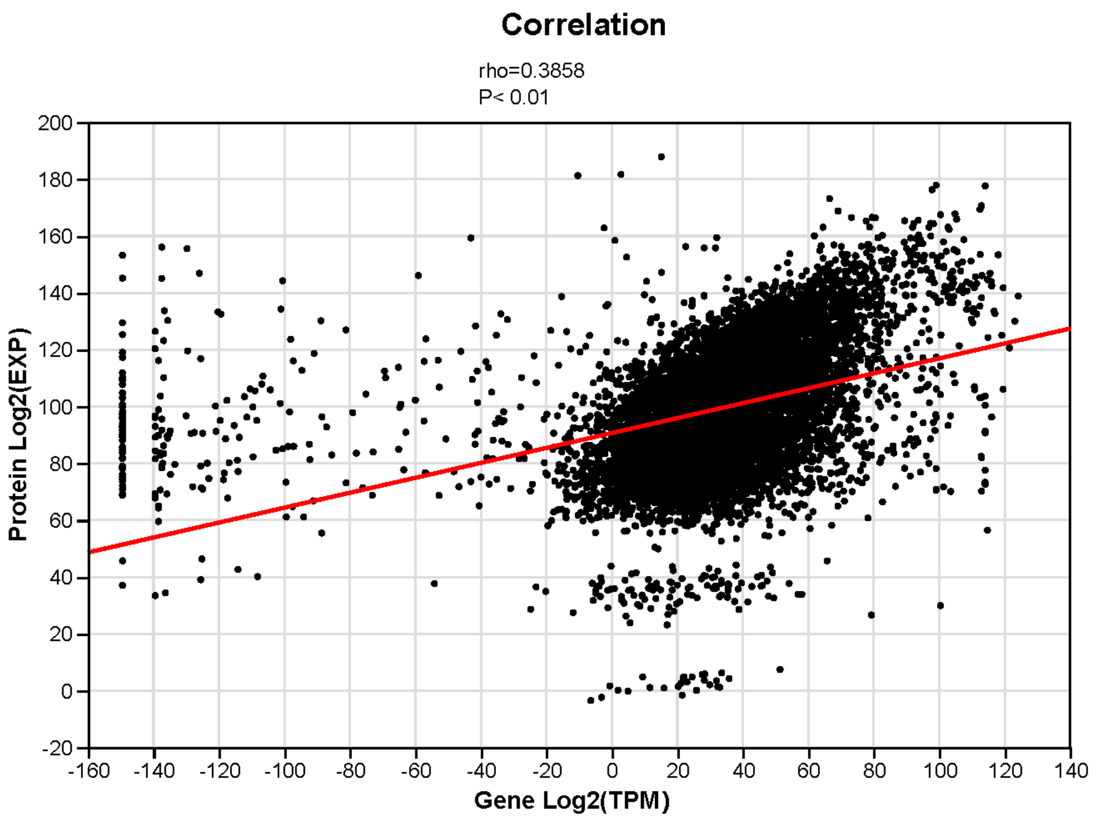

Figure S5. Following Venn-based intersection of the proteome and transcriptome datasets, a protein–transcript correlation was constructed for the resultant consensus gene set. Pearson correlation coefficients were then computed pairwise using the matched quantitative expression matrices from the proteomic and transcriptomic profiles.

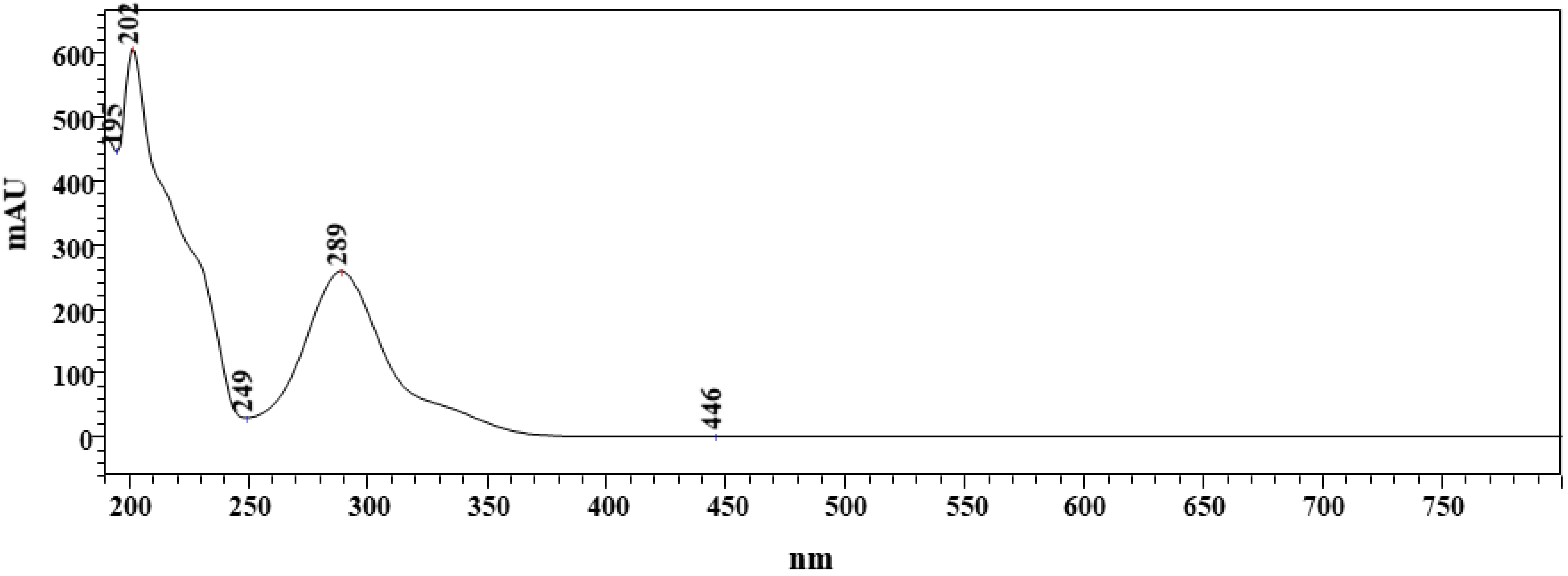

Figure S6. Full-spectrum HPLC analysis of taxifolin. The compound was scanned from 190–800 nm, revealing a prominent absorption peak at 289 nm, which overlaps the UVB region (280–320 nm).
